# Intimate partner violence and its associations among HIV-infected MSM with new drug abuse in Jinan, China

**DOI:** 10.1186/s12889-023-17451-4

**Published:** 2023-12-15

**Authors:** Yong Yu, Huiling Cai, Xi Chen, Fuqun Xiao, Keke Qin, Jiahong Li

**Affiliations:** 1 School of Politics and Public Administration, Guangxi Normal University, Guilin, 541006 Guangxi China; 2 Guangzhou Center for Disease Control and Prevention & Institute of Public Health, Guangzhou Medical University, Guangzhou, 510180 Guangdong China

**Keywords:** New Drugs, HIV-infected MSM, Dating Violence, Sexual risk behavior, ART medication adherence, Mental health

## Abstract

**Background:**

Intimate Partner Violence (IPV) is prevalent among HIV-infected men who have sex with men (MSM), with well-established risk factors and adverse outcomes. However, there is a lack of comprehensive investigation of both upstream risk factors and downstream adverse outcomes among HIV-infected MSM in a Chinese context. This study aimed to examine IPV and its associations among a Chinese sample of HIV-infected MSM.

**Methods:**

A cross-sectional study was conducted among 294 HIV-infected MSM in Jinan City from June to December 2020. All data were collected through an online questionnaire, which included IPV, sexual risk behavior, antiretroviral therapy (ART) adherence, depression, anxiety, and suicidal ideation. Chi-square tests and multivariate logistic regressions were performed to examine risk factors and adverse outcomes of IPV.

**Results:**

Of the 294 HIV-infected MSM, 71.1% experienced any IPV, including control (37.1%), threat of public identity (30.6%), emotional violence (25.2%), security threat (18.4%), and physical violence (13.9%). The prevalence of sexual risk behavior, good ART adherence, depression, anxiety, and suicidal ideation was 55.1%, 53.4%, 48.3%, 32.3%, and 65.0%, respectively. Abuse of methamphetamine (METH) (aOR:2.79; 95%CI:1.43 ~ 5.45), capsule 0 or stimulating liquid (aOR:2.68; 95%CI:1.31 ~ 5.47), Magu (aOR:3.16; 95%CI:1.51 ~ 6.60), and other new drugs (aOR:2.87; 95%CI:1.52 ~ 5.43), disclosing HIV infection to partners (aOR:2.03; 95%CI:1.10 ~ 3.78), and gay sexual orientation (aOR = 3.32; 95%CI: 1.82 ~ 6.05) were significantly correlated with the experience of IPV. In addition, IPV was significantly associated with sexual risk behavior (aOR = 2.02; 95%CI:1.16 ~ 3.53), ART adherence (aOR = 2.63; 95%CI:1.46 ~ 4.74), depression (aOR = 3.83; 95%CI:2.09 ~ 7.02), anxiety (aOR = 2.27; 95%CI:1.19 ~ 4.35), and suicidal ideation (aOR = 3.78; 95%CI:2.11 ~ 6.80).

**Conclusions:**

IPV is prevalent among HIV-infected MSM and is associated with poor behavioral and mental health, highlighting more efforts are needed to address this issue. The finding that new drug abuse, HIV disclosure, and gay sexual orientation are associated with increased risk of IPV provides essential insights for the development of comprehensive and targeted IPV prevention and intervention programs in the future.

## Background

Intimate Partner Violence (IPV) is a broad term that includes verbal, physical, emotional, and sexual abuse in a intimate relationship [1]. Globally, IPV is common in the general population, with a reported prevalence of 25–33% [2]. Men who have sex with men (MSM) are more likely to experience IPV than heterosexuals [3, 4]. A meta-analysis of 19 studies, mainly from the United States, reported that the incidence of IPV among MSM over the past five years was 32.0% (95%CI:19.32 ~ 44.58) [5]. A study on 1122 MSM in China showed that 9.8% (110/1122) had experienced IPV in the past 12 months [6]. Another study in China reported that the lifetime incidence of IPV among gay men was 32.8%, much higher than the reported 8.8% in heterosexual men [7]. HIV-infected MSM are even more likely to experience IPV than non-affected MSM [4]. A study in the United States showed that the risk of physical IPV among HIV-infected MSM was 50% higher than among MSM without HIV infection [8]. Another study reported that more than 1/4 of HIV-infected MSM (28.6%) had experienced IPV [9]. Given the widely prevalent IPV among HIV-infected MSM, it is imperative to understand its risk factors and adverse outcomes to guide further prevention and intervention programs on IPV.

Previous studies have identified a wide range of factors that contribute to the increased risk of IPV among HIV-infected MSM, among which the following three have been most widely reported: new drug abuse, HIV disclosure, and sexual orientation. New drugs, also known as “club drugs” and “recreational drugs”, mainly include products such as METH, Magu, cannabis, and ecstasy. They are characterized by strong psychological addiction, long duration of effects, and long half-life time, which may influence the behavior of the abuser and lead to reduced self-restraint and weakened integrity [10]. Compared with traditional drugs, new drugs result in higher sexual desire, more sexual partners, and more sexual risk behaviors in dating [10]. New drug addicts often have psychotic symptoms such as auditory hallucinations and hallucinations, which may lead to out-of-control behaviors and violence [11]. Although HIV disclosure has been proposed as an effective way to control HIV transmission through sex [12, 13], disclosing one’s HIV infection status may trigger violent reactions from dating partners [14–16]. A growing body of evidence has consistently demonstrated an increased incidence of IPV among HIV-infected MSM following HIV disclosure to their partners [14–16]. In addition, sexual orientation is another contributory factor of IPV that is unique to Chinese culture. In China, MSM are considered as having “psychological abnormalities” or “personal quality problems” and are highly discriminated [7]. A study [7] showed that Chinese gay men were 5.07 times more likely to experience IPV than heterosexual men after controlling for confounders. Specifically, MSM often face identity disclosure threats, with a study showing that 12.4% of MSM have been threatened to disclose their sexual orientation [7].

In addition to identifying the upstream risk factors of IPV, it is equally crucial to investigate the downstream adverse outcomes related to IPV among HIV-infected MSM. A growing body of evidence has indicated that the experience of IPV may result in adverse effects on the behaviors and mental health of HIV-infected MSM, including sexual risk behaviors, ART nonadherence, and poor mental health. Previous studies have consistently demonstrated positive associations between IPV and sexual risk behaviors such as inconsistent condom use and multiple sexual partners [17, 18]. Multiple mechanisms may explain the link between IPV and sexual risk behaviors, including substance abuse and depression following IPV and the threat of disclosing one’s sexual orientation or HIV infection status during IPV, which may all increase sexual risk behaviors [17, 18]. There is also ample evidence showing that IPV may interfere with antiretroviral therapy (ART), which can inhibit the virus replicating, thus increasing the risk of continued HIV transmission [19, 20]. A meta-analysis found that IPV was significantly associated with lower ART use, poorer self-reported ART adherence, and lower odds of viral load suppression [21]. The hindering effect of IPV in ART initiation and adherence may be explained by several plausible mechanisms, including fear of new and continued IPV, partner control, feelings of denial and shame, and poor mental health, which may all inhibit access to HIV care [19–21]. Finally, the strong link between IPV and poor mental health, such as depression and anxiety, has been well established by extensive studies. Several recent literature reviews have shown significant associations between experience of IPV and adverse mental health outcomes, including posttraumatic stress disorder, depression, anxiety, and suicidal ideation and attempts [22–24]. A critical conceptual framework to understand the association between IPV and poor mental health is the minority stress theory, which posits that experiences of external prejudice events such as discrimination and victimization may produce internal stress processes such as internalized homophobia and concealment, which further contribute to elevated risk of psychological distress [23].

**Fig. 1:**
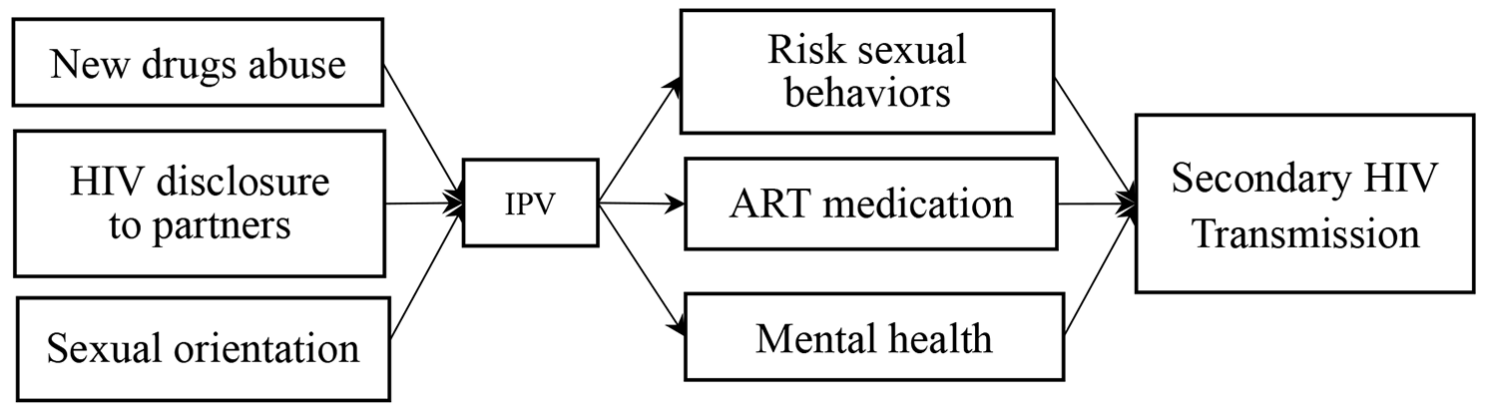
Potential pathway of Secondary HIV Transmission of HIV-infected MSM with New Drug Abuse MSM

Although the above-mentioned risk factors and adverse outcomes of IPV have been extensively reported in the literature, most previous studies focused on only one or two aspects of these factors, and there is a lack of comprehensive and continuous investigation of both the upstream risk factors and downstream adverse outcomes of IPV. In addition, the bulk of IPV studies targeted female victims in the general population and were conducted in Western countries; much less is known about IPV and its associations among HIV-infected MSM in a Chinese context where both HIV and MSM are highly discriminated against. To address the research gaps, we conducted the current study to systematically examine the prevalence, risk factors, and adverse outcomes of IPV in a sample of Chinese HIV-infected MSM. Our study will provide essential insights into IPV among HIV-infected MSM and guide the development and implementation of intervention and prevention programs to prevent IPV. Based on the review of previous studies, we drew a conceptual map of IPV and its associations to guide our study, as shown in Fig. 1. We proposed the following hypotheses:

### Hypothesis 1

New drug use, HIV disclosure, and sexual orientation are risk factors of IPV among HIV-infected MSM.

### Hypothesis 2

HIV-infected MSM who have experienced IPV are at increased risk of risk sexual behaviors, ART nonadherence, and poor mental health.

## Methods

### Study design and participants

This cross-sectional study was conducted in the HIV counseling and testing clinic of the Center for Disease Control and Prevention (CDC) of the Central District of Jinan City from June to December 2020. HIV-infected MSM confirmed in the CDC were enrolled as continuous samples. The inclusion criteria were: (1) self-reported MSM; (2) HIV-infected and had started ART; (3) self-reported use of any of the following new drugs in the past six months: METH, Magu, cannabis, ecstasy, capsule 0 or stimulating liquid, topping e-cigarettes, ketamine, and happy water. (4) Had a partner (spouse in marriage, fixed sex partner, casual sex partner) and had sex with them in the past six months. (5) Age ≧ 18 years old; (6) Currently living in the city; (7) Voluntarily participated in the study and signed the informed consent form. Exclusion criteria: (1) those who had a partner in the past six months but did not have sex with them; (2) those who were unable to complete the study due to severe physical or mental illness. A total of 310 participants met the criteria for enrollment during the sample recruitment period, among whom 294 participants completed the study with a valid questionnaire, with a response rate of 94.8%. The sample size aligns with the standard recommended by the World Health Organization for sentinel surveillance of sexually transmitted infections among high-risk populations (250 ~ 400) [7].

### Data collection

The study was reviewed and approved by the Ethics Committee of Guangxi Normal University (GXNU [2020] 2002). All participants provided written informed consent before participating in the study. Participants were approached by a clinic nurse and referred to our investigators, who then made an appointment to conduct an on-site questionnaire survey with the participants. Due to the sensitive contents of the survey, all data were collected through an online survey on Sojump (http://www.sojump.com), one of China’s most popular online survey platforms. Sojump is a WeChat-based survey program that provides professional online research services such as questionnaire design, distribution, data collection, data analysis, and result reporting [25]. Before filling out the online questionnaire, the investigator showed the paper version of the informed consent form to the respondents, who then read and signed it. In order to protect the privacy of participants, all questionnaires were self-administered by the respondents on Sojump using our designated iPads. Participants filled in the online questionnaire in the designated office without any interruption and left the survey site after finishing the questionnaire. During the questionnaire survey, no survey instructor was involved to ensure the anonymity and privacy of the survey as far as possible and to obtain true and reliable data.

### Measurements

#### IPV

IPV was assessed using the Dating Violence Questionnaire (DVQ) [7], which investigates the following six types of IPV in recent six months: (a) control: your partner (married spouse, fixed sexual partner, temporary sexual partner) has tried to control most of your daily activities. For example, you cannot go out or talk to anyone; (b) emotional violence: your partner always scolds you, and you feel embarrassed or sad about it; (c) security threat: your partner has threatened you because of anger, and you are worried about your safety; (d) physical violence: your partner has hurt your body, including but not limited to punches and kicks, burns, stabs, etc.; (e) sexual violence: your partner has sexually assaulted you, or forced to have sex with you when you are reluctant; (f) threat of public identity: your partner has threatened to tell others that you are gay or bisexual, an HIV/AIDS patient or a drug addict.

#### New drug abuse

Participants were asked to indicate whether they had used any of the following new drugs in the past six months: METH, Magu, cannabis, ecstasy, capsule 0 or stimulating liquid, topping e-cigarettes, ketamine, and happy water. Each drug with a positive answer about uses within the past six months was coded as “1” and “0” for no use. In this study, Rush (isoamyl nitrite, referred to as Rush in MSM) was not included in the category of new drugs. Rush is very popular among MSM in China, with a reported abuse rate of as high as 92.4% among MSM [26]. The inclusion of Rush in the category of new drugs may lead to an excessively high proportion of drug users in the MSM population. Besides, Rush can temporarily enhance sexual pleasure and effectively relieve pain during anal sex and is considered harmless by most MSM [10]. The Chinese judiciary has not listed Rush as an illegal drug, so it is still controversial to include Rush as a new drug.

#### Sexual risk behavior

Participants were asked to indicate whether they had engaged in the following four types of sexual risk behaviors in the past six months: (a) commercial sex (having sex for money or goods); (b) temporary sex (single-sex, non-commercial sex); (c) group sex (3 or more people having sex together); and (d) condomless anal intercourse (CAI). Participants with a positive answer to any of the above four behaviors were considered as having sexual risk behaviors and coded as “1”, while those who replied “no” to all of the four behaviors were regarded as having no sexual risk behaviors and coded as “0”.

#### HIV disclosure

Participants were asked to indicate whether they had disclosed their HIV status to any of the following partners: (a) the spouse (if married); (b) the fixed sexual partner (sexual partner with whom the participant has the highest frequency of sex excluding the spouse); (c) the most recent temporary partner. Participants who disclosed their HIV status to any of the above three types of partners were coded as “1” and “0” otherwise.

#### ART medication adherence

ART medication adherence was assessed using the Center for Adherence Support Evaluation (CASE) [27], developed based on the results of correlation analysis and principal component analysis by previous studies. The CASE included the following three items: (a) Overall, the condition of not being able to take antivirals on time ____ occurs. (i) every time; (ii) most of the time; (iii) occasionally; (iv) never. (b) On average, how many days per week does a missed medication occur? (Missed dose of medication defined as ≥ 1 missed dose a day) (i) daily; (ii) 4–6 days; (iii) 2–3 days; (iv)1 day; (v) < 1 day per week on average; (vi) never; (c)When was the last time you missed taking antiviral drugs? (i)in the last week; (ii)1 to 2 weeks ago; (iii)3 to 4 weeks ago; (iv)1 to 3 months ago; (v)3 months ago; (vi)never. The total CASE score ranges from 3 to 16, with a higher score indicating a higher level of adherence and a CASE index > 10 meaning good ART medication adherence.

#### Mental health

This study investigated three types of mental problems: depression, anxiety, and suicidal ideation. Participants’ anxiety and depression symptoms in the past two weeks were assessed by the Chinese versions of the Patient Health Questionnaire (PHQ-2) [28] and Generalized Anxiety Disorder (GAD-2) [29], respectively. The total scores of both PHQ-2 and GAD-2 range from 0 to 6, with a cutoff of 3 distinguishing between those with and without depression/anxiety. In this study, both scales demonstrated good internal consistency with Cronbach α coefficients of 0.85 and 0.88, respectively. Beck Scale for Suicide Ideation-Chinese Version (BSI-CV) [30]was used to evaluate the subject’s suicidal ideation in the past week or at the height of depression. The scale consists of 19 items under two domains: suicidal ideation (the first five items) and suicidal tendency (the last 14 items). A higher score indicates stronger suicidal ideation and a higher risk of suicide. In this study, suicidal ideation was assessed by the following two items: item 4: “How much do you want to try to commit suicide?” and item 5: “To what extent do you want external forces to end your life, that is, to have a ‘passive suicidal desire’? (for example, hoping to sleep forever and never wake up, die accidentally, etc.)”. Participants who answered “weak” or “moderate to strong” to either item were defined as having suicidal ideation. BSI-CV has shown good reliability and validity in China [26]. In this study, BSI-CV showed good internal consistency with a Cronbach α of 0.86.

#### Control variables

We collected information on the following indicators as control variables: age, household registration, ethnicity, marital status, education level, monthly income, the length since HIV diagnosis, comorbidity with other chronic diseases, and the recent physical examination results.

### Data analysis

Frequencies and proportions were used to present categorical variables, while means and standard deviations (SDs) were used to describe continuous variables. Chi-square tests were used to evaluate factors associated with IPV. To explore the risk factors of IPV, we conducted several multivariate logistic regressions with IPV as the dependent variable and use of each new drug, HIV disclosure, and sexual orientation as the independent variables while adjusting for all control variables. To investigate the adverse outcomes related to IPV, we performed several multivariate logistic regressions with IPV as the independent variable and sexual risk behavior, ART adherence, depression, anxiety, and suicidal ideation as the dependent variables, respectively, while adjusting for all control variables.

All analysis was conducted by SPSS 22.0 (SPSS, Inc., Chicago, IL, USA).

## Results

### Sample characteristics, key indicators, and associations with IPV

A total of 294 HIV-infected MSM were included in the final analysis. Tables 1 and 2 show the sample characteristics and key indicators. Participants had a mean age of 33.0 (SD:7.3) years, with an average length of 36.5 (SD:25.4) months since HIV diagnosis. Most participants were gay men (61.2%), of Han nationality (93.9%), not married (83.0%), living in cities (61.2%), had bachelor’s or above education (42.2%), and had a monthly income of > 4000 Yuan (60.5%). Over one-third of participants were comorbid with other chronic diseases (35.7% ), and most participants (78.6%) had normal results in their last physical examinations.

**Table 1:** Experience of IPV among respondents with different characteristics [n (%)]

Variables		Sample size (n /%) n = 294	Experienced IPV n = 209	Not experienced IPV n = 85	*χ* ^2^	*P–values*
Sexual orientation	Gay men	177(60.2)	146(82.5)	31(17.5)	28.11	<0.001
	Bisexual men	117(39.8)	63(53.8)	54(46.2)		
Length of time for HIV-positive diagnosis/months	≤ 42	161(54.8)	110(68.3)	51(31.7)	1.32	0.250
	>42	133(45.2)	99(74.4)	34(25.6)		
Age/years	≤ 33	173(58.8)	124(71.7)	49(28.3)	0.07	0.79
	>33	121(41.2)	85(70.2)	36(29.8)		
nationality	Han nationality	276(93.9)	197(71.4)	79(28.6)	0.18	0.669
	Ethnic minority	18(6.1)	12(66.7)	6(33.3)		
Marital status	Unmarried/divorced/widowed	244(83.0)	173(70.9)	71(29.1)	0.02	0.876
	Married	50(17.0)	36(72.0)	14(28.0)		
Place of household registration	City	180(61.2)	120(66.7)	60(33.3)	4.42	0.036
	Rural areas	114(38.8)	89(78.1)	25(21.9)		
Education level	Below bachelor’s degree	124(42.2)	86(69.4)	38(30.6)	0.31	0.575
	Bachelor degree or above	170(57.8)	123(72.4)	47(27.6)		
Monthly income/Yuan	≤ 4000	116(39.5)	84(72.4)	32(27.6)	0.16	0.686
	>4000	178(60.5)	125(70.2)	53(29.8)		
Have any other chronic diseases other than HIV	Yes	105(35.7)	72(68.6)	33(31.4)	0.50	0.478
	No	189(64.3)	137(72.5)	52(27.5)		
Are all indicators normal in the last medical examination	Yes	231(78.6)	170(81.3)	61(26.4)	3.29	0.070
	No	63(21.4)	39(61.9)	24(38.1)		
Abuse METH	Yes	122(41.5)	97(79.5)	25(20.5)	7.19	0.007
	No	172(58.5)	112(65.1)	60(34.9)		
Abuse Magu	Yes	101(34.4)	86(85.1)	15(17.6)	14.80	<0.001
	No	193(65.6)	123(63.7)	70(36.3)		
Abuse capsule 0 or stimulating liquid	Yes	92(31.3)	77(83.7)	15(16.3)	10.36	0.001
	No	202(68.7)	132(65.3)	70(34.7)		
Abuse other new drugs	Yes	144(49.0)	114(79.2)	30(20.8)	8.96	0.003
	No	150(51.0)	95(63.3)	55(36.7)		
HIV notification to partners	Yes	149(50.7)	120(80.5)	29(19.5)	13.12	<0.001
	No	145(49.3)	89(61.4)	56(38.6)		
ART medication adherence	Good	157(53.4)	96(61.1)	61(38.9)	16.20	<0.001
	Poor	137(46.6)	113(82.5)	24(17.5)		
Whether there is risk sexual behavior	Yes	162(55.1)	127(78.4)	35(21.6)	9.37	0.002
	No	132(44.9)	82(62.1)	50(37.9)		
Depression	Yes	142(48.3)	119(83.8)	23(16.2)	21.60	<0.001
	No	152(51.7)	90(59.2)	62(40.8)		
Anxiety	Yes	95(32.3)	78(82.1)	17(17.9)	8.23	0.004
	No	199(67.7)	131(65.8)	68(34.2)		
Suicidal idea	Yes	191(65.0)	153(80.1)	38(19.9)	21.56	<0.001
	No	103(35.0)	56(54.4)	47(45.6)		

**Table 2:** Presents the frequency distribution of key indicators for IPV, risky sexual behavior, ART medication adherence, and mental health among respondents (n = 294)

Indexes	*n* (%)	Indexes	*n* (%)
**Experienced IPV**	209(71.1)	**Have risk sexual behavior**	162(55.1)
Control	109(37.1)	Casual sexual behavior	114(38.8)
Threat of public identity	90(30.6)	CAI	95(32.3)
Threatened by others to disclose being HIV-infected	41(13.9)	Group sexual behavior	87(29.6)
Threatened by others to be openly gay or bisexual	36(12.2)	Commercial sexual behavior	33(11.2)
Threatened by others to disclose being a drug addict	28(9.5)	Buying sex	28(9.5)
Emotional violence	74(25.2)	Selling sex	11(3.7)
Security threat	54(18.4)	**Mental health**	
Physical violence	41(13.9)	Depression	142(48.3)
Sexual violence	29(9.9)	Anxiety	95(32.3)
**Good ART medication adherence**	157(53.4)	Suicidal ideation	191(65.0)

In the past six months, 71.1% (209/294) of participants experienced at least 1 type of IPV, with control as the most common type (37.1%), followed by threat of public identity (30.6%), emotional violence (25.2%), security threat (18.4%), and physical violence (13.9%). All participants had abused new drugs in the past six months, with METH (41.5%) as the most frequently reported specific new drug. Approximately half of the participants (50.7%,145/294) had disclosed their HIV infection to their partners. When asked about the reasons for non-disclosure, 53.8% (78/145) were concerned about conflict and relationship breakdown, and 40.0% (58/145) were concerned about potential physical harm (data not shown in the table). Over half of the participants (55.1%, 162/294) had engaged in sexual risk behaviors, with casual sex as the most common type (38.8%), followed by CAI (32.3%), group sex (29.6%), and commercial sex (11.2%). The average score of the CASE index was 12.0 ± 4.0, and 53.4% of the participants had good ART medication adherence. The prevalence of depression, anxiety, and suicidal ideation was 48.3%, 32.3%, and 65.0%, respectively.

In the univariate analysis of *χ*^2^ tests, variables significantly correlated with experience of IPV in the prior six months (*P* < 0.05) were sexual orientation, household registration, abuse of each type of new drug, HIV disclosure to partners, ART medication adherence, sexual risk behavior, depression, anxiety, and suicidal ideation. Details are shown in Table 1.

### Risk factors of IPV

Table 3 shows the results of the multivariate analysis of the risk factors of IPV. After controlling for other confounders, abuse of METH (aOR = 2.79; 95%CI: 1.43 ~ 5.45), capsule 0 or stimulating liquid (aOR = 2.68; 95%CI: 1.31 ~ 5.47), Magu (aOR = 3.16; 95% CI:1.51 ~ 6.60), and other new drugs (aOR = 2.87; 95%CI:1.52 ~ 5.43), disclosing HIV infection to partners (aOR = 2.03; 95%CI: 1.10 ~ 3.78), and sexual orientation (aOR = 3.32; 95%CI: 1.82 ~ 6.05) were significantly correlated with the experience of IPV, supporting hypothesis 1.

**Table 3:** Multifactorial analysis with IPV of respondents as the dependent variable*

	Independent variable		*β*	*SE*	*Waldχ* ^*2*^	*aOR(*95%*CI)*	*P–values*
Model 1	Abuse METH	Yes	1.03	0.34	9.08	2.79(1.43 ~ 5.45)	0.003
		No				1	
	Abuse Magu	Yes	0.99	0.37	7.28	2.68(1.31 ~ 5.47)	0.007
		No				1	
	Abuse capsule 0 or stimulating liquid	Yes	1.15	0.38	9.34	3.16(1.51 ~ 6.60)	0.002
		No				1	
	Abuse other new drugs	Yes	1.06	0.33	10.55	2.87(1.52 ~ 5.43)	0.001
		No				1	
	HIV disclosure to partners	Yes	0.71	0.32	5.05	2.03(1.10 ~ 3.78)	0.025
		No				1	
	Sexual orientation	gay men	1.20	0.31	15.37	3.32(1.82 ~ 6.05)	<0.001
		Bisexual men					

*Note:The parameter beta of the regression model is estimated while controlling for the following factors: age, household registration, ethnicity, marital status, education level, monthly income, the duration since HIV diagnosis, comorbidity with other chronic diseases, and the recent physical examination results

### Adverse outcomes of IPV

Table 4 shows the results of the multivariate analysis of adverse outcomes of IPV. After controlling for other factors, IPV was significantly correlated with sexual risk behavior (aOR = 2.02; 95%CI:1.16 ~ 3.53), ART medication adherence (aOR = 2.63; 95%CI: 1.46 ~ 4.74), depression (aOR = 3.83; 95%CI:2.09 ~ 7.02), anxiety (aOR = 2.27; 95%CI:1.19 ~ 4.35), and suicidal ideation (aOR = 3.78; 95%CI:2.11 ~ 6.80), supporting hypothesis 2. Finally, all our results supported the conceptual map outlined in Fig. 1.

**Table 4:** Multivariate analysis of HIV disclosure, risk sexual behavior, ART medication adherence, depression, anxiety, suicidal ideation as dependent variables and IPV as independent variables *

	Dependent variables		Independent variables		*β*	*SE*	*Waldχ* ^*2*^	*aOR(*95%*CI)*	*P*
Model 2	Risk sexual behavior	Yes	IPV	yes	0.70	0.28	6.15	2.02(1.16 ~ 3.53)	0.013
		No		no				1	
Model 3	ART medication adherence	Poor		yes	0.97	0.30	10.39	2.63(1.46 ~ 4.74)	0.011
		Good		no				1	
Model 4	Depression	Yes		yes	1.34	0.31	18.82	3.83(2.09 ~ 7.02)	< 0.001
		No		no				1	
Model 5	Anxiety	Yes		yes	0.82	0.33	6.10	2.27(1.19 ~ 4.35)	0.013
		No		no				1	
Model 6	Suicidal ideation	Yes		yes	1.33	0.30	19.81	3.78(2.11 ~ 6.80)	< 0.001
		No		no				1	

* Note:The parameter beta of the regression model is estimated while controlling for the following factors: age, household registration, ethnicity, marital status, education level, monthly income, the duration since HIV diagnosis, comorbidity with other chronic diseases, and the recent physical examination results

## Discussion

To our knowledge, this is the first study to comprehensively investigate the prevalence, risk factors, and adverse outcomes of IPV among a Chinese sample of HIV-infected MSM, based on a conceptual map drawn from previous studies. Our study showed an alarmingly high prevalence of IPV among HIV-infected MSM, with 71.1% experiencing at least one IPV in the past six months, including 13.9% of physical violence and 9.9% of sexual violence. These results were significantly higher than those reported in a previous study conducted among 418 gay men in Guangzhou, China, using the same questionnaire to measure IPV, which showed that 32.8% had experienced IPV, including 7.9% of physical violence and 9.6% of sexual violence [7]. One primary reason for the higher prevalence of IPV in the current study is that all participants were new drug users, as drug use is a well-established risk factor for IPV. In addition, the discrepancies in IPV prevalence reported in various studies may be explained by the differences in investigation time, location, and sample characteristics. Our findings suggest that IPV is a common and severe issue among HIV-infected MSM who use new drugs, and there is an urgent need to develop intervention and prevention programs to alleviate IPV.

Identifying risk factors of IPV is crucial for developing targeted interventions to prevent IPV. Consistent with previous studies [31, 32], our study showed that new drug use, HIV disclosure, and gay sexual orientation were associated with an increased risk of IPV among HIV-infected MSM. The association between the use of various types of new drugs and IPV may be related to the characteristics of the new drugs, including strong addiction and long-lasting effects. The strong link between new drug abuse and IPV may be explained by the increased sexual compulsivity following new drug use [33]. Specifically, new drug use may lead to increased sexual fantasies and exaggerated expressions of sexual behavior, such as group sex, CAI, temporary sex, commercial sex, sexually sadistic and masochistic behavior, and fisting behavior, which all contribute to violent injuries in sexual behavior.

The positive association between HIV disclosure to partners and IPV is congruent with previous studies showing increased IPV following HIV disclosure [14–16]. Although disclosing one’s HIV status to partners has been proposed as an effective strategy to control HIV transmission through sex [12, 13], it may also increase the risk of potential IPV [11]. Instead of receiving care and social support, disclosing HIV infection via a sexual route to a partner, especially a spouse, may lead to relationship breakdown and even IPV, as it indicates cheating and betraying [34]. In China, drug use, HIV infection, and MSM are considered socially unacceptable and thus highly discriminated against; HIV-infected MSM who are drug users may conceal their identities and rarely disclose to others due to the triple stigma and fear of losing their social reputation and values [13]. As shown in our study, only half of the participants disclosed their HIV infection to their partners. HIV disclosure may increase the risk of being blackmailed during conflicts, as evidenced in our study that a large proportion of participants had been threatened by their partners to disclose their status of HIV infection, sexual orientation, or drug use. It is noteworthy that violence and identity threats may, in turn, impede HIV disclosure, indicating a bidirectional relationship between HIV disclosure and IPV [11].

Our study showed that sexual orientation was another factor associated with experiencing IPV, with gay men being over three times more likely to experience IPV than bisexual men. This may be explained by the different social acceptance of gay men and bisexual men in a Chinese social-cultural context. In China, many people still judge whether a person’s sexual orientation is normal by whether they have a girlfriend or are married and have children. Bisexual men are more likely to have heterosexual sex, enter heterosexual marriages, and conceal their sexual orientations, with studies showing that about a quarter of MSM were married, and more than half of unmarried MSM had marriage intentions [35, 36]. Therefore, compared to bisexual men, gay men have higher perceived stigma and are thus more vulnerable to IPV in romantic relationships. Another explanation may be the different sexual roles of gay men and bisexual men in the male-male relationship. Previous research showed [37] that most bisexual men acted as the insertive or active side in the male-male relationship, while bisexual men acted as the passive side. Similar to the male-female relationship where a female partner, as the passive side, tends to be the victim of IPV, gay men are also more likely to experience IPV.

In addition, our study found that experience of IPV was associated with increased risk of sexual risk behavior, ART nonadherence, depression, anxiety, and suicidal ideation, which aligns with the bulk of literature showing IPV leading to a series of behavioral and mental disorders [17, 18, 22].

The positive association between IPV and sexual risk behaviors may be related to the victims’ fear of new and continued violence if they refuse unsafe sex or suggest condom use. IPV victims are often in a passive position in terms of negotiating condom use, sexual initiation, and sexual exclusivity [17]. HIV-infected MSM who experienced IPV may forgo condom use in serious dating relationships, such as pro-couples and couples, due to concern of revealing their HIV status by insisting on condom use [14]. The finding that HIV-infected MSM had poor ART adherence was consistent with previous studies showing a lack of access and adherence to HIV care in other populations experiencing IPV [11]. Partner control and emotional distress, such as fear, denial, and depression, may explain the link between IPV and poor ART adherence [19, 20] ART adherence directly determines the effectiveness of ART, and poor adherence may lead to the failure of viral suppression, increasing the probability of opportunistic infections and drug resistance [38]. Finally, our study showed that IPV was associated with poor mental health, including depression, anxiety, and suicidal ideation, which were all prevalent among HIV-infected MSM experiencing IPV. Previous studies [39, 40] have also confirmed that IPV can, to some extent, cause severe and persistent mental health problems. HIV-infected MSM are a highly discriminated group, and the experience of IPV may further exaggerate their minority stress, leading to emotional distress and mental disorders [23]. Furthermore, cognitive impairment caused by some psychiatric disorders can significantly impair the judgment and ability of HIV-infected MSM to adhere to safe sex practices, leading to increased risk of CAI, multiple partners, and drug abuse [41–43].

### Limitation

This study has the following limitations. First, the cross-sectional study design precludes any causal inference. Second, the hidden nature of the study population indicated that random sampling cannot be conducted. Thus, the representativeness and the extrapolation of the study sample were limited. Third, all data were collected based on self-report, which may be subject to recall bias and social desirability bias. Fourth, we did not collect information about the time of violence, the number of perpetrators, and the frequency of violence. Fifth, due to China’s current crackdown on illegal behavior, such as drug abuse and IPV, our survey on these sensitive issues may result in underreporting and information bias.

## Conclusions

In conclusion, IPV is prevalent among HIV-infected MSM, highlighting more clinical, research, and political efforts are needed to address this issue. New drug use, HIV disclosure, and sexual orientation are risk factors for experiencing IPV among HIV-infected MSM, which, in turn, are associated with an increased risk of sexual risk behavior, ART nonadherence, and mental health problems such as depression, anxiety, and suicidal ideation. Our findings provide important insights for the development of comprehensive and targeted IPV prevention and intervention programs in the future. Some valuable strategies may include integrating primary IPV prevention interventions, establishing routine IPV screening, and deploying related services for victims of violence.

## Data Availability

The datasets generated and/or analyzed during the current study are not publicly available but are available from the corresponding author upon reasonable request.
