# Correction: Intimate partner violence and its associations among HIV-infected MSM with new drug abuse in Jinan, China

**DOI:** 10.1186/s12889-024-17937-9

**Published:** 2024-02-14

**Authors:** Yong Yu, Huiling Cai, Xi Chen, Fuqun Xiao, Keke Qin, Jiahong Li

**Affiliations:** 1 School of Politics and Public Administration, Guangxi Normal University, 541006 Guilin, Guangxi China; 2 Guangzhou Center for Disease Control and Prevention & Institute of Public Health, Guangzhou Medical University, 510180 Guangzhou, Guangdong China

**BMC Public Health (2023) 23:2517**

10.1186/s12889-023-17451-4

**Incorrect**

Huilin Cai

**Correct**

Huiling Cai

